# Norwegian citizens’ responses to influxes of asylum seekers: comparing across two refugee crises

**DOI:** 10.1080/15534510.2023.2242619

**Published:** 2023-08-26

**Authors:** Lise Bjånesøy, Hege H. Bye

**Affiliations:** a Department of Government, University of Bergen, Bergen, Norway; b Department of Psychosocial Science, University of Bergen, Bergen, Norway

**Keywords:** Asylum seekers, refugees, immigration attitudes, intergroup behavior, Ukraine, Syria

## Abstract

We compared Norwegians’ attitudes to immigration, perspective taking, and intergroup behaviors directed at asylum seekers in 2016 (Syrians and Afghans) and 2022 (Ukrainians). We find evidence for a stronger exclusionary response to the asylum seekers in 2016 than in 2022. Attitudes to immigration were more negative in 2016 than in 2022, and skepticism and avoiding asylum seekers was more common. However, the dominant behavior in both years was prosocial (greeting and donating) and Norwegians’ willingness to take asylum seekers perspective was similar in 2016 and 2022. These results may reflect an absence of a symbolic threat in 2022 and may be connected to differences in the political rhetoric about asylum seekers in 2015/2016 and 2022.

Within less than a decade, Europeans in many countries have experienced two large influxes of asylum seekers. In 2015, more than 1 million people sought refuge in Europe, many due to the civil war in Syria (Eurostat, 2016). In 2022, following Russia’s invasion of Ukraine, 7.8 million people have fled from Ukraine to other European countries (UNHCR, 2022). It is already established that the political responses to these two crises have been markedly different, both in Norway and the EU. Whereas Ukrainians are granted collective temporary protection (Ministry of Justice and Public Security, 2022; The Council of the European Union, 2022), Syrian (and other) asylum seekers have their applications assessed on an individual basis. Populist radical right parties have softened their hard stance against asylum seekers and expressed support for Ukrainian refugees (Albertazzi et al., 2022). In Norway, the leader of the Progress Party, Sylvi Listhaug, argued that Ukrainians should be exempt from the introduction program that other asylum seekers are required to complete, emphasizing that ‘there is no need for an introduction to Norwegian culture or an extensive introduction to Norwegian society. A lot is similar [to Ukraine]’ (Røsvik, 2022). There is also evidence to suggest that initial media portrayals of people fleeing from Ukraine were less negative than those of people fleeing from Syria and other countries in the Middle East and North Africa (Zawadzka-Paluektau, 2022). Differences such as these have led some researchers to argue that there is a pervasive double standard in the treatment of refugees based on their country of origin and race (Freedman et al., 2022). What has received less attention is differences in citizen attitudes and behavioral responses across these two crises.

In this brief report we compare Norwegian citizens’ attitudes to immigration, perspective taking, and intergroup behaviors directed at asylum seekers in response to the 2015/2016 influx of primarily Syrians and Afghans and the influx of Ukrainians in 2022. Empirically, we draw on data collected in the Norwegian Citizen Panel shortly after both influxes. This provides a unique opportunity to compare public responses to asylum seekers with identical measures fielded as real-world events were unfolding. This comparative approach complements and extends previous research on attitudes to asylum seekers and refugees (Bansak et al., 2016; Bjånesøy, 2019; Cowling et al., 2019; Nordø & Ivarsflaten, 2022).

## Background

There are several studies of Europeans’ responses to the 2015/2016 refugee crisis. Reactions among citizens in transit and destination countries in Europe in 2015/2016 were mixed. On the one hand, some studies showed neutral or marginally exclusionary reactions in neighborhoods hosting asylum seekers (Deiss-Helbig & Remer, 2021), reduced right-wing voting among voters in contexts where meaningful intergroup contact was possible (Steinmayr, 2021), and that community members’ negative expectations to asylum seekers did not materialize, leading to increased acceptance (Bygnes, 2020). On the other hand, studies also showed that general attitudes towards immigrants became more negative, that citizens preferred more restrictive policies, and that there was an increase in voting for right-wing parties among exposed voters (Dinas et al., 2019; Hangartner et al., 2019; Nordø & Ivarsflaten, 2022; Steinmayr, 2021). These latter studies document exclusionary reactions.

After Russia’s invasion of Ukraine in February 2022 and the resulting mass flight, public debate about differences in the reception of refugees across the two crises soon arose. Not only were European political responses and media portrayals different (Dražanová & Geddes; Zawadzka-Paluektau, 2022), but the question of differential reactions on part of the receiving populations quickly emerged. Anecdotal evidence points to a warmer welcome in Norway of Ukrainians in 2022 as compared to the predominately Syrian and Afghan asylum seekers in 2015/2016 (Apeland, 2022). However, extensive documentation of citizens’ responses to the influx of Ukrainians, and how that compares to the responses in 2015/2016, has not yet emerged in the scientific literature. In 2016, Bansak et al. 2016 studied Europeans’ attitudes to asylum seekers. They included both Syrian, Afghan and Ukrainian profiles in their conjoint design and did not find that Europeans were more accepting of Ukrainians than Syrians at that point in time. After the Russian invasion, Dražanová and Geddes (2022) performed a survey experiment in eight European countries, directly comparing support for accepting Syrian and Ukrainian refugees to the country. Support for accepting Ukrainian refugees were higher in all eight countries, especially in Central and Eastern Europe. Together, these two studies may suggest that a preference for accepting Ukrainian asylum seekers have developed among Europeans after the Russian invasion.

De Coninck (2022) discusses differences in the reception of Ukrainian and Afghan refugees in Europe. Consistent with previous research documenting differential reactions to immigrants based on race/ethnicity and religious background (Esses, 2021), he argues that Ukrainians benefit from being predominately White and Christian, circumventing the symbolic-threat reactions (Stephan & Stephan, 2000) facing Afghans and Muslims. We know that the influx of asylum seekers in 2015 (temporarily) shifted immigration attitudes in the Norwegian population in a negative direction before returning to pre-crisis levels 18 months later (Nordø & Ivarsflaten, 2022). Following from De Coninck’s (2022) reasoning, we would expect Norwegian citizens’ attitudes to immigration to be more negative in 2016 compared to 2022.

Central to De Coninck’s (2022) argument, and the broader public discussion (Bayoumi, 2022), is the notion that, for Europeans, Ukrainians are ingroup members (Tajfel & Turner, 1979). Ingroup members are more likely to be targets of prosocial behaviors (e.g., greetings, friendship, help) and less likely targets of skepticism, avoidance, and harm (Cuddy et al., 2007). Willingness to take the perspective of asylum seekers (i.e., ‘the active cognitive process of imagining the world from another’s vantage point or imagining oneself in another’s shoes to understand their visual viewpoint, thoughts, motivations, intentions, and/or emotions.’ (Ku et al., 2015, p. 79)) may also be facilitated by a shared ingroup membership. Although the link between perspective taking and prosocial behavior is not straightforward (Sassenrath et al., 2022), perspective taking has been linked to inclusive behavior towards refugees (Adida et al., 2018). We explore the prosocial and avoidant or harming behaviors directed at asylum seekers in 2016 and 2022, as well as Norwegians’ willingness to take asylum seekers’ perspective at these two points in time.

### The Norwegian context

In 2022, the Norwegian Directorate of Immigration registered 36.228 applications for protection from Ukrainian citizens, women and children comprising the vast majority of applicants (The Norwegian Directorate of Immigration UDI, 2023). In 2015, the total number of asylum applications was 31.150, of which 10.448 applicants were Syrians and 7000 Afghans. Men comprised the majority of applicants among both Syrians and Afghans (The Norwegian Directorate of Immigration UDI, 2016). To put these numbers into perspective, the total number of asylum applications filed with Norwegian immigration authorities the years before the two influxes of asylum seekers was 11.480 in 2014 (The Norwegian Directorate of Immigration UDI, n.d.) and 1656 in 2021 (The Norwegian Directorate of Immigration UDI, 2022).

After the first asylum seekers started entering Norway during the 2015 refugee crisis the government and the political parties in parliament quickly agreed to make asylum policies stricter with the aim of trying to decrease the number of asylum applicants. This broad agreement resulted in an asylum deal with 18 actions to restrict access to Norway and prevent asylum seekers from coming (Johansen, 2015; Stortinget [The Storting], 2016). All parties^1^ except The Socialist Left Party and the Green Party supported the agreement.

The political context during the arrivals of asylum seekers from Ukraine in 2022 was markedly different. Instead of tightening asylum policies, there was broad agreement to welcome the Ukrainian refugees, and some of the consequences of the war in Ukraine was explicitly put on the Norwegian government’s budged, such as humanitarian and military aid (The Norwgian Government, 2022). Instead of preventing asylum seekers from coming, there was broad agreement to help the Ukrainian refugees. The anti-immigrant party the Progress Party (FrP) stated that they would stop refugees from other countries and rather prioritize Ukrainian refugees, as they share the same European culture (The Progress Party, 2022b). The party also suggested to increase border control to prevent unwelcome refugees to exploit the more generous system (The Progress Party, 2022a). Given these very different political contexts, a general expectation would be that citizen responses to asylum seekers were more positive in 2022 than in 2016 (Esses, 2021; Gaucher et al., 2018).

## Method

### Participants and procedure

The data we present in this paper were collected in November 2016 (wave 7) and in May and June 2022 (wave 24) using the research-infrastructure the Norwegian Citizen Panel (NCP; Ivarsflaten et al., 2016a, 2016b). The NCP is a research-purpose internet panel where potential respondents were identified based on random samples from the national population registry and invited to take part in an online survey two to three times a year. All participants have provided their written informed consent before participating in the panel. The NCP follows the EU General Data Protection Regulation (GDPR) and a Data Protection Impact Assessment (DPIA) has been conducted in cooperation with Sikt – Norwegian Agency for Shared Services in Education and Research. The DPIA number is 118,868 (DIGSSCORE, 2023). As detailed in the measures section, our analyses focus on three measures: 1) a question targeting general attitudes to immigration, 2) five items addressing behaviors directed at asylum seekers, and 3) a question about taking the perspective of asylum seekers.

In wave 24 (Ivarsflaten et al., 2022), the questions about attitudes to immigration, behaviors towards asylum seekers, and perspective taking were asked to the entire panel (*N* = 10 160). In wave 7 (Ivarsflaten et al., 2016b), the question about attitudes to immigration was asked to a random subpanel (*n* = 1203) and the questions about behaviors towards asylum seekers were asked to a second random subpanel of respondents (*n*  = 1103). Finally, the question about perspective taking were asked to both these subpanels (*n* = 2306). One question about donating to asylum seekers was not included in wave 7. Instead, we employed a question from wave 6 (March and April 2016; Ivarsflaten et al., 2016a) that was included in a module on volunteering and had a very similar wording. This question was asked to a random subpanel of respondents (*n* = 1190).

As documented in the methodology reports for each wave of the NCP (Skjervheim & Høgestøl, 2016a, 2016b; Skjervheim et al., 2022), weights need to be applied for the NCP samples to match the population characteristics in terms of gender, age, geography and education. Unless otherwise noted, we present weighted estimates of all statistics, employing survey weights calculated for the respective survey wave. The NCP has run since 2013 and new respondents have been recruited several times (waves 1, 3, 8, 11, 14, 16, 18, and 22). This means that some, but not all, respondents have participated in several waves of data collection. Of the participants who answered the immigration attitude question in wave 24, 565 were also in the subpanel who answered this question in wave 7. For perspective taking, 1085 answered the question in both wave 7 and wave 24. The behaviors directed at asylum seekers were reported by 529 and 570 respondents across wave 24 and 7 and 6, respectively. We will take advantage of this panel structure by including analyses of change over time among respondents with repeated measures. In Table 1, we present an overview of survey years, panels, and number of respondents.

**Table 1.** Overview of survey years, panels, *N*, and missing data.

	Year and Survey Wave			
	2022 (wave 24)	2016 (wave 7)	2016 (wave 7)	2016 (wave 6)
Panels (NCP subpanel numbers)	1, 2, 3, 4and 5	4	3	1
*N*	10160	1203	1103	1190
**Question**	**Missing cases (‘No answer’)**			
Advantage or disadvantage that immigrants come to live here?	34	17		
Greeted some of the asylum seekers	412		60	
Become friends with some of the asylum seekers	452		76	
Expressed skepticism to asylum seekers to others	462		78	
Avoided the asylum seekers as far as possible	467		79	
Donated money, clothing or equipment	325			12
Thought about or imagined what it is like to arrive in Norway as an asylum seeker?	96	25	35	

### Measures

#### Attitudes to immigration

We measured citizens’ attitudes towards immigration with the question ‘In your opinion how great an advantage or disadvantage is it for Norway that immigrants come to live here?’ The respondents answered on a seven-point scale ranging from ‘very great disadvantage’ to ‘very great advantage’.

#### Behaviors directed at asylum seekers

To capture behaviors directed at asylum seekers, the respondents read the following introduction ‘Norway has received many asylum seekers recently. People have had different experiences of this. What have your experiences been? I have … ’ followed by items describing different behaviors. Specifically, respondents indicated whether they had ‘Greeted some of the asylum seekers’, ‘Become friends with some of the asylum seekers’, ‘Expressed skepticism towards asylum seekers to others’, and ‘Avoided the asylum seekers as far as possible’. Response categories were ‘Yes’, ‘No’, and ‘Not relevant’. In 2022 the item ‘Donated money, clothing or equipment’ was also included in the list. This was not included in the list in 2016 (wave 7, November). Instead, we included the following item from wave 6 (March/April): ‘Many refugees and asylum seekers have come to Norway in the course of the last year. During the last 12 months, have you made any of the following contributions in connection with the refugee situation? Donation (e.g., money/clothing/equipment)’.

#### Perspective taking

Perspective taking was measured by the item ‘To what extent have you thought about or imagined what it is like to arrive in Norway as an asylum seeker?’ The respondents answered on a five-point scale ranging from ‘Not at all’ to ‘a very great extent’.

### Statistical analyses

Our statistical analyses consist of two parts for each of the three measures. We first present means (immigration attitudes, perspective taking) or percentages (behaviors directed at asylum seekers) with 95% confidence intervals employing survey weights to describe and compare attitudes, behaviors and perspective taking in the adult Norwegian population in 2016 and 2022. Second, we present paired samples *t*-tests to assess change over time in attitudes and perspective taking among subsets of respondents for whom we have repeated measures in 2016 and 2022. For the behavioral items, we repeat the analyses of proportions engaging in each behavior among the respondents who answered in both waves. In these second parts of the analyses, we employ unweighted data.

As shown in Table 1, there were very few missing responses for the attitude to immigration and perspective taking questions (from 0.3% to 2.6%), and analyses were carried out without imputing missing values. There were more missing responses across the behavioral items (from 1.0% to 7.2%). In order not to overestimate the proportions of respondents who indicated a behavior (i.e., answered ‘yes’), proportions were calculated based on the total *n* of respondents who were asked a question.

## Results

### General immigration attitudes

On average, general attitudes to immigration were more positive (*M* = 4.70, *SD*  = 1.36, 95% CI [4.67, 4.73]) in 2022 than in 2016 (*M* = 4.10, *SD*  = 1.42, 95% CI [4.03, 4.18]). Among respondents who answered the attitude to immigration question in both 2016 and in 2024 (*n* = 565), a paired samples *t*-test on unweighted data showed that attitudes to immigration had shifted in a positive direction from 2016 (*M* = 4.32, *SD*  = 1.44) to 2022 (*M* = 4.74, *SD*  = 1.35), *t*(546) = − 10.10, *p* < .001, Cohen’s *d*_*z*_ = −0.43. These results show a substantive change both in the overall population means and over time within individuals.

**Figure 1.**
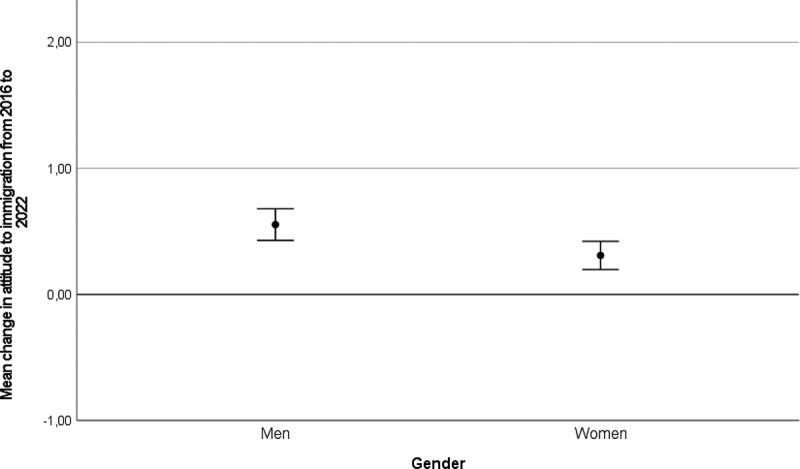
Change in attitude to immigration from 2016 to 2022 by gender.

**Figure 2.**
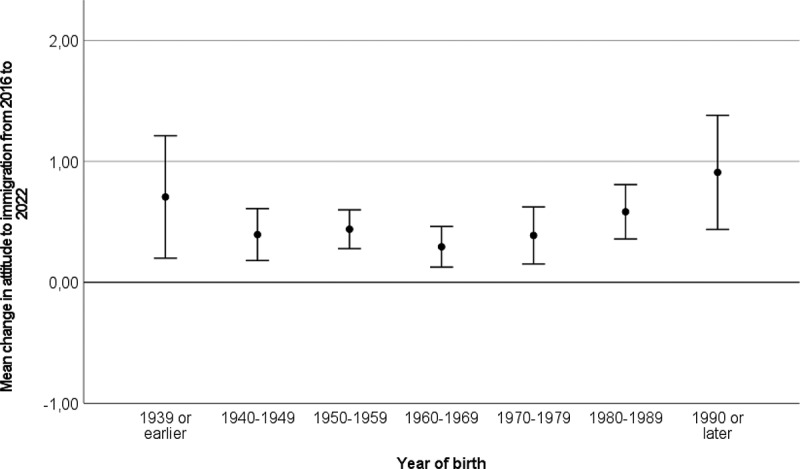
Change in attitude to immigration from 2016 to 2022 by age group.

**Figure 3.**
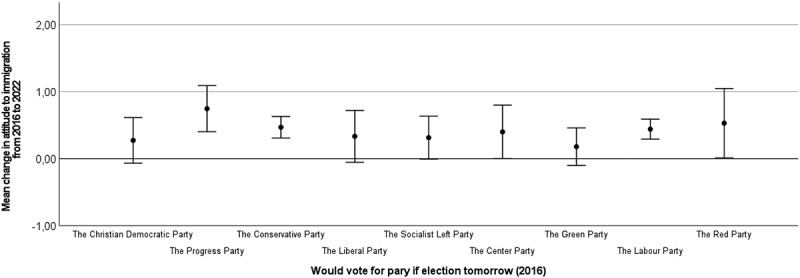
Change in attitude to immigration from 2016 to 2022 by preferred political party.

To probe this change in attitude to immigration further, we calculated change scores at the individual level (i.e., subtracting the attitude score in 2016 from the score in 2022. A score of 0 indicates no change, negative scores indicate more negative attitudes to immigration in 2022 than in 2016, positive scores indicate a more positive attitude to immigration in 2022 than in 2016. Range −4 to 4). In our sample (*n* = 565), 65.8% of the respondents had changed their response from 2016 to 2022. As illustrated in Figures 1 through 3, a change in the direction of more positive attitudes to immigration from 2016 to 2022 is present across gender (Figure 1), age groups (Figure 2), and intentions to vote for different political parties in 2016 (Figure 3). What we observe is that the change in a positive direction is present across demographic segments of the sample and is not driven by a narrow sub-group.

Error bars represent 95% confidence intervals. 0 on the y-axis represents no change.

Error bars represent 95% confidence intervals. 0 on the y-axis represents no change.

Error bars represent 95% confidence intervals. 0 on the y-axis represents no change.

### Behaviors directed at asylum seekers

**Figure 4.**
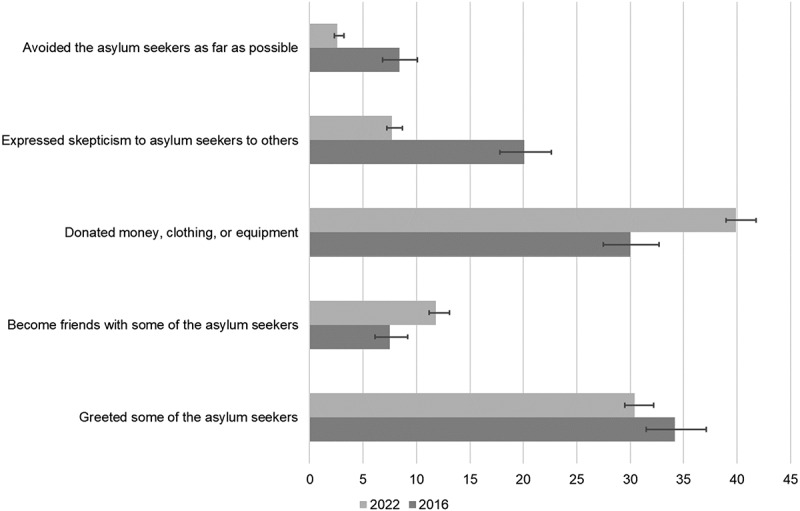
Behaviors directed at asylum seekers in 2016 and 2022. Percentage of respondents indicating a behavior.

In Table 2 and Figure 4, we present the percentage of respondents who said ‘yes’ to the items describing behaviors directed at asylum seekers. Across the five behaviors, we find that larger proportions of respondents reported befriending asylum seekers and donating money, clothing or equipment in 2022 than in 2016. We also find that in 2022, smaller proportions of respondents reported expressing skepticism or avoiding asylum seekers than in 2016. As an exception to this broader pattern of more prosocial and less skepticism and avoidant behavior in 2022 than in 2016, we found that the proportion of respondents indicating having greeted asylum seekers was slightly higher in 2016 as compared to 2022. In both 2016 and 2022 greeting (34.2% and 30.4%) and donating (30.0% and 39.9%) were the most common behaviors. The largest difference across the two years was in expressed skepticism which was indicated by 20.1% in 2016 and 7.7% in 2022.

Percentages are based on weighted data to estimate population proportions.

**Table 2.** Behaviors directed at asylum seekers in 2016 and 2022. Percentage of respondents indicating a behavior.

	Year			
	2016		2022	
Behavior	%	95% CI	%	95% CI
Greeted some of the asylum seekers	34.2	[31.5, 37.1]	30.4	[29.5, 31.3]
Become friends with some of the asylum seekers	7.5	[6.1, 9.2]	11.8	[11.2, 12.5]
Donated money, clothing, or equipment	30.0	[27.5,32,7]	39.9	[39.0, 40.9]
Expressed skepticism to asylum seekers to others	20.1	[17.8, 22.6]	7.7	[7.2, 8.2]
Avoided the asylum seekers as far as possible	8.4	[6.8, 10.1]	2.6	[2.3, 2.9]

Percentages are based on weighted data to estimate population proportions.

**Figure 5.**
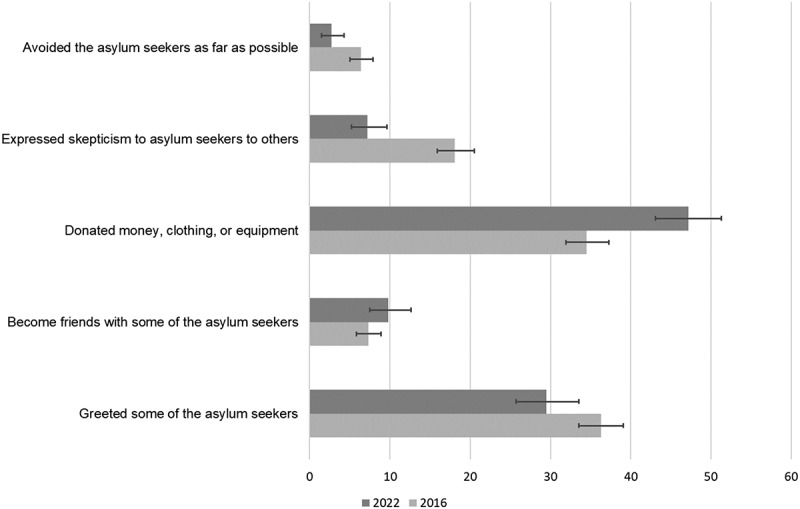
Behaviors directed at asylum seekers in 2016 and 2022 among respondents who participated in both waves. Percentage of respondents indicating a behavior.

The analyses focusing exclusively on the behaviors reported by respondents who answered the behavioral items in both 2016 and 2022 are presented in Table 3 and Figure 5. These results largely mirror the results presented in Table 2 and indicate less avoidance, less expressed skepticism, and more donations in 2022 than in 2016. Of those who reported expressing skepticism in 2016, 74.1% said ‘no’ or indicated that it was not relevant in 2022. Similarly, 78.8% percent of those who indicated avoidance in 2016, said ‘no’ or ‘not relevant’ in 2022.

Percentages are based on unweighted data as respondent weights are not identical across panel waves.

**Table 3.** Behaviors directed at asylum seekers in 2016 and 2022 among respondents who participated in both waves. Percentage of respondents indicating a behavior.

	Year			
	2016		2022	
Behavior	%	95% CI	%	95% CI
Greeted some of the asylum seekers	36.3	[33.5, 39.1]	29.5	[25.7, 33,5]
Become friends with some of the asylum seekers	7.3	[5.8, 8.9]	9.8	[7.5, 12.6]
Donated money, clothing, or equipment	34.5	[31.9, 37.3]	47.2	[43.1, 51.3]
Expressed skepticism to asylum seekers to others	18.1	[15.9, 20.5]	7.2	[5.2, 9.6]
Avoided the asylum seekers as far as possible	6.4	[5.0, 7.9]	2.7	[1.5, 4.3]

Percentages are based on unweighted data as respondent weights are not identical across panel waves.

### Perspective taking

We find no evidence that Norwegians were more ready to take asylum seekers’ perspective in 2022 (*M* = 3.10, *SD*  = 0.96, 95% CI [3.08, 3.12]) than in 2016 (*M* = 3.05, *SD*  = 1.01, 95% CI [3.01, 3.09]). Among those respondents who answered the perspective taking question in both 2016 and in 2022 (*n* = 1085), a paired samples *t*-test on unweighted data showed no significant change in perspective taking from 2016 (*M* = 3.13, *SD*  = 0.97) to 2022 (*M* = 3.12, *SD*  = 0.90), *t*(1084) = 0.39, *p* = .698, Cohen’s *d*_*z*_ = 0.01.

## Discussion

We compared Norwegian Citizens’ attitudes to immigration, perspective taking, and intergroup behaviors directed at asylum seekers in 2016 (primarily Syrians and Afghans) and 2022 (Ukrainians). First, we find evidence for a stronger exclusionary response to the asylum seekers in 2016 than in 2022. Attitudes to immigration were more negative in 2016 than in 2022 in the Norwegian population, and among respondents with repeated measures, attitudes had shifted in a positive direction from 2016 to 2022. Behaviorally, skepticism and avoiding asylum seekers was more common in 2016 than in 2022. Most respondents who indicated expressing skepticism or avoidance in 2016 (∼ 74 and 78%) did not indicate these behaviors in 2022. These findings are consistent with previous research documenting preferences for culturally close and White immigrants, Christians, and women (Bansak et al., 2016; Esses, 2021; Ford, 2011) and supports De Coninck’s (2022) argument that Ukrainians are less likely to trigger symbolic threat reactions among Europeans.

At the same time, we also find that the dominant response in both years was prosocial; the most commonly reported behaviors were greeting asylum seekers and donating money, clothing or equipment. Having greeted some of the asylum seekers was reported by approximately 1 in 3 citizens in both years (34.2% in 2016 and 30.4% in 2022). Friendships were less common (7.5% in 2016 and 11.8 in 2022), and like the pattern for donations (30.0% in 2016 and 39.9% in 2022) showed a slight preference for Ukrainians. These latter results are in line with a preference for Ukrainians as ingroup members (Cuddy et al., 2007; Tajfel & Turner, 1979). However, there may also have been structural causes such as a more developed infrastructure for donations in 2022 and a faster introduction of Ukrainians into Norwegian schools, universities, and workplaces. We did not find that Norwegians’ willingness to take asylum seekers perspective was higher in 2022 than in 2016, and respondents with repeated measures showed no change over time. This latter result is inconsistent with the idea that Ukrainian asylum seekers were viewed through the lens of a shared ingroup membership. Thus, although we do find some evidence for a more welcoming reception of Ukrainians (friendships, donations) than asylum seekers who arrived in 2015/2016, the key difference between citizens’ reactions is less hostility as indicated by more positive immigration attitudes and less expressed skepticism in 2022.

Our study cannot speak directly to the causes of the differences observed across 2016 and 2022. However, our conjecture is that both more positive media frames and the absence of rhetorical attacks on Ukrainians have been important for shaping citizen responses. From previous research, we know that how immigrants are constructed in the media and political rhetoric matters for public perceptions (Esses, 2021; Gaucher et al., 2018; Verkuyten, 2004). Media frames employed during the 2015 crisis were dominated by security threats and economization (i.e., emphasizing the economic costs; Greussing & Boomgaarden, 2017; Zawadzka-Paluektau, 2022). Although empirical analyses of the media coverage of the mass flight from Ukraine is still limited, there is evidence that Ukrainian refugees are not portrayed with the negative frames used to describe MENA refugees (Zawadzka-Paluektau, 2022). Similarly, the dramatic shift in rhetoric by traditionally anti-immigrant politicians is particularly telling (Albertazzi et al., 2022). The absence of a threat narrative may have contributed to a lack of a threat response in the population in 2022. It is also possible that differences in public perceptions have roots in the nature of the situations people are fleeing from: an invasion by an aggressive neighboring country may be perceived differently from an internal civil war.

We recognize that there are also other differences across the two crises that we have studied that may have impacted our results. Whereas the influx of Ukrainian refugees was ongoing when our data were collected, the data from 2016 were collected at a point in time where the influx was very recent but not ongoing due to the closing of boarders across Europe. It is possible that the differences we observed in friendships and donations might grow over time as contact between Norwegians and Ukrainian asylum seekers develop further. Following the development of attitudes and intergroup relations over time is an important task for future research, especially if the political rhetoric changes (e.g., financial costs become politicized). Empirical investigations of the causal mechanisms behind the differences we document would also be a relevant avenue for future work. That said, this study provides an important first step in empirically documenting similarities and differences in citizen’s attitudes and behaviors towards asylum seekers in the two largest refugee crises in Europe since World War II. We have shown that differences across time are not clear cut. It is the absence of skepticism – not the absence of prosocial behaviors – that appear to be the main difference in how asylum seekers were received among Norwegians.

## Data Availability

The data used in this study is available in online repositories, however, they are openly shared in single waves. In this paper, data across three waves (6, 7, and 24) were combined using respondent identification codes. These data will be made available for research and replication purposes, however, to protect participants’ anonymity, they are only available via a restricted access secure server. Please contact digsscore@uib.no for questions regarding data access.

## Note

1. The parties that supported the agreement were The Labor Party, The Conservative Party, The Center Party, The Progress Party, The Christian Democratic Party, and The Liberal Party.

## open-scholarship open-materials

This article has earned the Center for Open Science badge for Open Materials. The materials are openly accessible at Norwegian Citizen Panel (NCP) Wave 6: https://doi.org/10.18712/NSD-NSD2344-V7, NCP Wave 7: https://doi.org/10.18712/NSD-NSD2401-V6, and NCP Wave 24: https://doi.org/10.18712/NSD-NSD3056-V1
